# Safety of Levetiracetam in Paediatrics: A Systematic Review

**DOI:** 10.1371/journal.pone.0149686

**Published:** 2016-03-01

**Authors:** Oluwaseun Egunsola, Imti Choonara, Helen Mary Sammons

**Affiliations:** Division of Medical Sciences and Graduate Entry Medicine, School of Medicine, University of Nottingham, Royal Derby Hospital, Derby, DE22 3DT, United Kingdom; Iowa State University, UNITED STATES

## Abstract

**Objective:**

To identify adverse events (AEs) associated with Levetiracetam (LEV) in children.

**Methods:**

Databases EMBASE (1974-February 2015) and Medline (1946-February 2015) were searched for articles in which paediatric patients (≤18 years) received LEV treatment for epilepsy. All studies with reports on safety were included. Studies involving adults, mixed age population (i.e. children and adults) in which the paediatric subpopulation was not sufficiently described, were excluded. A meta-analysis of the RCTs was carried out and association between the commonly reported AEs or treatment discontinuation and the type of regimen (polytherapy or monotherapy) was determined using Chi^2^ analysis.

**Results:**

Sixty seven articles involving 3,174 paediatric patients were identified. A total of 1,913 AEs were reported across studies. The most common AEs were behavioural problems and somnolence, which accounted for 10.9% and 8.4% of all AEs in prospective studies. 21 prospective studies involving 1120 children stated the number of children experiencing AEs. 47% of these children experienced AEs. Significantly more children experienced AEs with polytherapy (64%) than monotherapy (22%) (p<0.001). Levetiracetam was discontinued in 4.5% of all children on polytherapy and 0.9% on monotherapy (p<0.001), the majority were due to behavioural problems.

**Conclusion:**

Behavioural problems and somnolence were the most prevalent adverse events to LEV and the most common causes of treatment discontinuation. Children on polytherapy have a greater risk of adverse events than those receiving monotherapy.

## Introduction

Levetiracetam (LEV) is one of the most commonly prescribed new generation antiepileptic drugs (AEDs). It accounts for about 8% of paediatric AED prescriptions in the UK [1] and 7% in Germany [2] and Hong Kong [3]. It was approved for adult use in Europe in 2000 and subsequently for paediatric use in 2005[4]. In the UK, it is recommended as an adjunctive agent for partial seizures, myoclonic seizures as well as for some epilepsy syndromes such as: rolandic epilepsy and late-onset childhood occipital epilepsy [5]. Some of the adverse reactions commonly associated with LEV include: somnolence, dizziness and behavioural problems. Behavioural problems are more commonly reported in children than adults [6].

LEV undergoes elimination predominantly by renal excretion. Metabolism by hydrolysis and hydroxylation is a minor process. This is thought to reduce the risk of drug-drug interactions [7]. Its onset of action is rapid, which makes it a candidate drug for the treatment of status epilepticus [8].Levetiracetam is approved for use in children as adjunctive therapy. It can be prescribed as monotherapy to adolescents 16 years old and over. Although, the safety of the drug in children and adolescents less than 16 years old is unknown, monotherapy use in this population is quite common [6].

Due to the increasing utilisation of LEV in children, this systematic review aims to evaluate the available evidence, from all studies, on the safety of LEV in children.

## Method

This review was carried out as per PRISMA guidelines. The systematic review protocol was not published

### Search strategy

Databases EMBASE (1974- February 2015) and Medline (1946-February 2015) were searched for articles in which paediatric patients (≤18 years) received LEV treatment for epilepsy. The paediatric search terms used were: paediatric* or pediatric* or boy* or girl* or pediatric* or child* or neonat* or infan* or adolescen* or newborn* or baby or toddler or young, in title [9]. This was combined with levetiracetam, also in title. Output was limited to humans and journal articles. Only studies that evaluated and reported safety outcomes were included. Papers published in English, Chinese, French and Spanish were included. Studies involving adults, mixed age population (i.e. children and adults) in which the paediatric subpopulation was not sufficiently described, were excluded.

### Data extraction

The types and number of AEs were extracted. Other extracted data included: the age of the patients, the dose, route of administration, the number of study participants, co-administered drugs, type of regimen (monotherapy or polytherapy) and the duration of follow up. Adverse reactions resulting in the discontinuation of LEV treatment were also documented

### Data quality assessment

The quality of the RCTs were assessed using the Cochrane collaboration’s tool for assessing risk of bias in randomised trials [10]. The quality of the prospective observational studies were assessed using the System for the Unified Management of the Review and Assessment of Information (SUMARI)[11]. Data from any study fulfilling 4 or more of the 9 criteria were included in the final data aggregation (S1 appendix). All studies were independently assessed by two reviewers. Any conflicting outcomes were discussed between the reviewers before a verdict was agreed upon.

### Data collection and statistical analysis

A meta-analysis of the RCTs was done using Revman version 6. Only AEs identified from 2 or more studies were analysed. The relative risks of AEs present in at least two RCTs were calculated, with a RR>1 suggesting that more AE were associated with LEV treatment. The data were considered homogeneous I^2^ ≤50% or Chi^2^ p≥0.05. Homogeneous data were analysed using the fixed effect model while the random effect model was used for heterogeneous data. Risk of AEs per 100 patient was also determined in both prospective and retrospective studies. Chi^2^ analysis of the association between each AE and the treatment regimen (polytherapy or monotherapy) was performed. Chi^2^ analysis of the association between treatment discontinuation and the treatment regimen was also conducted. P-values <0.05 were considered statistically significant for all analyses.

## Results

### Description of study characteristics

**Fig 1:**
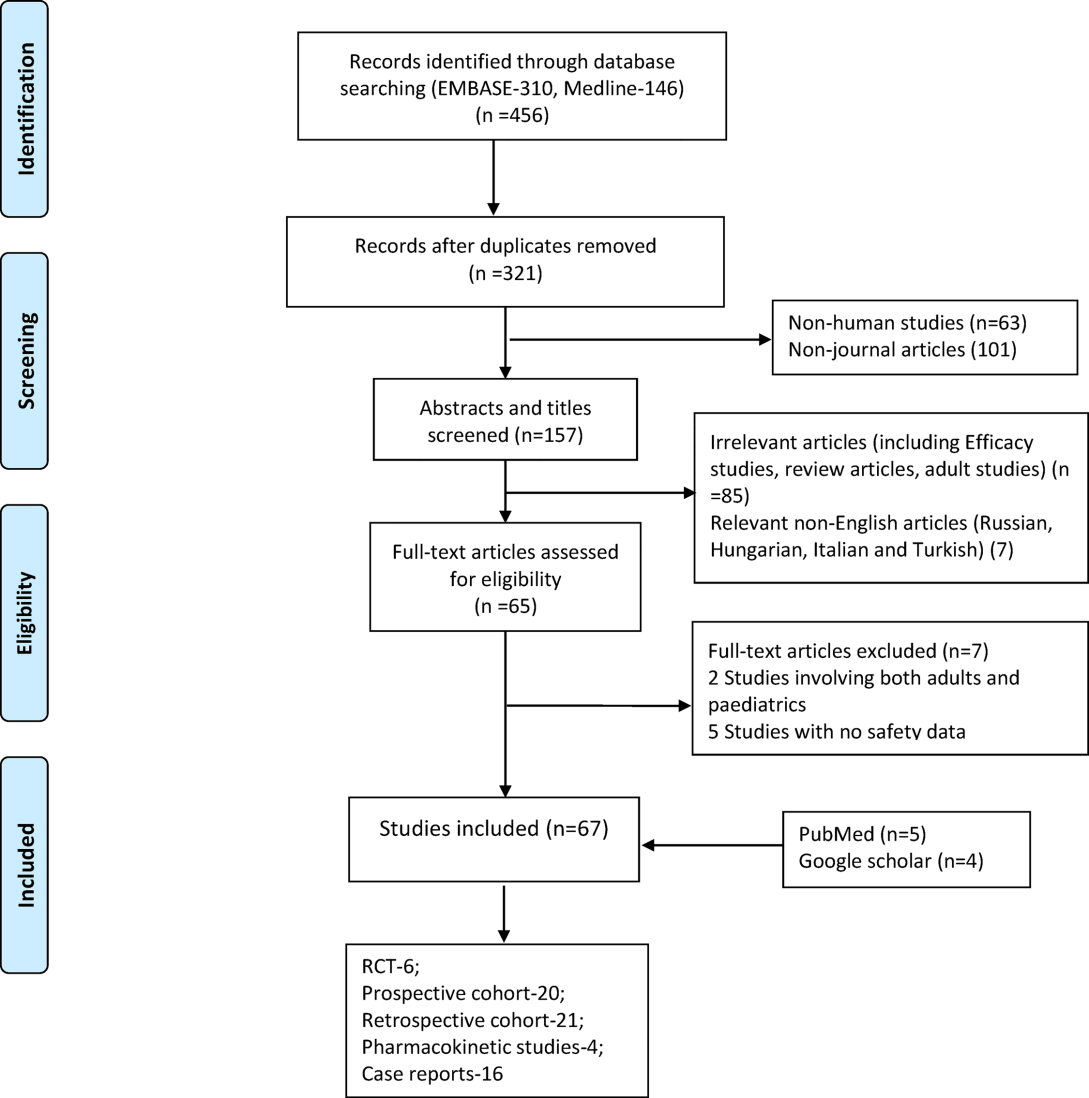
Flow chart of included studies.

**Fig 2:**
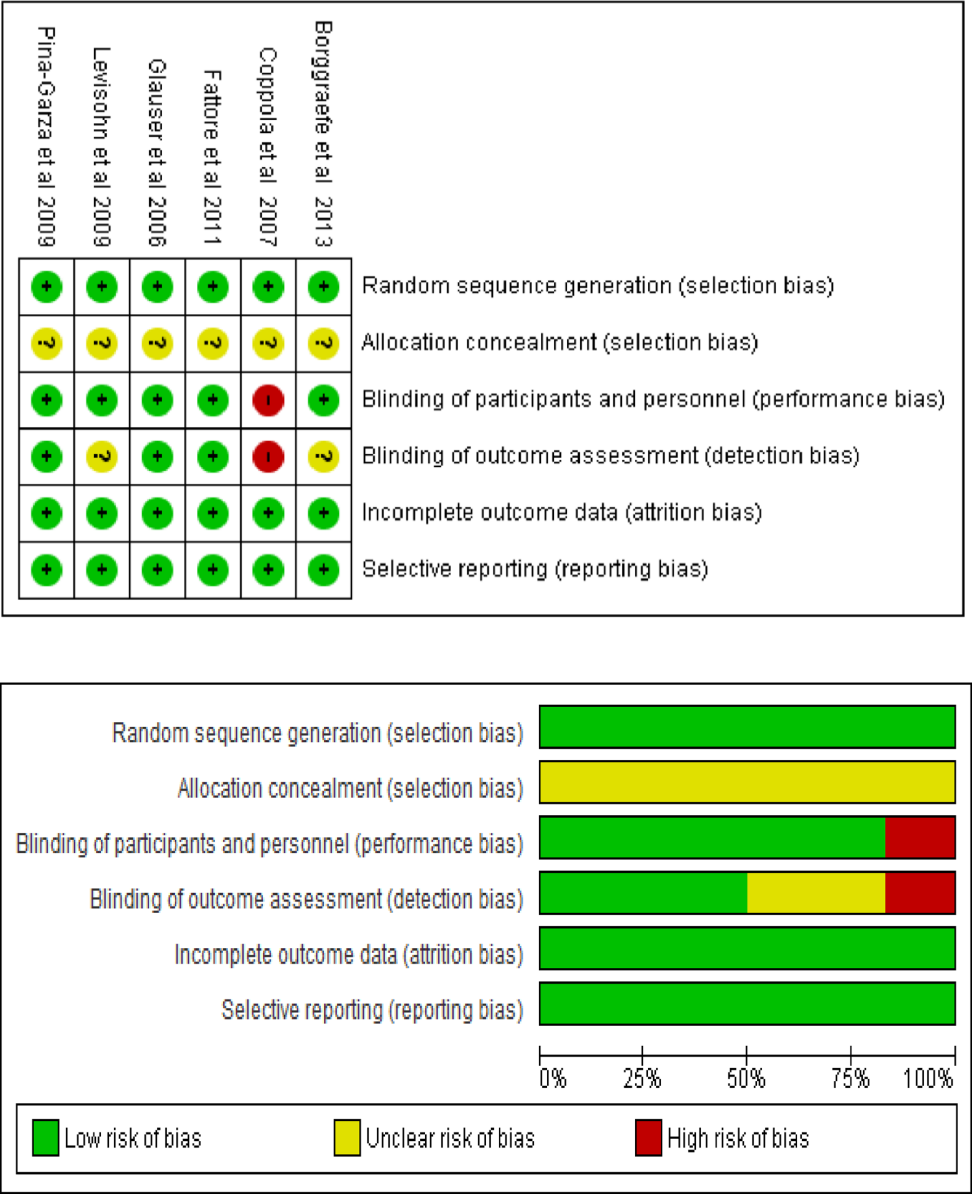
Risk of bias summary and graph.

Sixty seven articles involving 3,174 paediatric patients were identified (Fig 1). No RCT or prospective cohort study was excluded after quality analysis (Fig 2). Quality analysis was not performed for retrospective studies. A total of 1,913 AEs were reported across studies. The largest numbers of children (1,461) were recruited within the 21 retrospective studies (Table 1). The greatest number of AEs (897) however was reported in the 20 prospective cohort studies. There were 6 RCTs with 415 reports of AEs.

**Table 1:** Summary of included studies.

Study type	Number of studies	Number of AEs (%)	Number of patients (%)
RCTs	6	415	306 (10%)
Prospective cohort studies	20	897	1311 (41%)
Pharmacokinetic studies	4	32	73 (2%)
Retrospective cohort studies	21	546	1461 (46%)
Case reports	16	23	23 (1%)
Total	**67**	**1913**	**3174**

The median period of follow-up was 24 weeks [IQR: 16.5–51 weeks]. Levetiracetam was administered orally in the majority of the studies (32 studies). In 8 studies, it was given intravenously; while both IV and oral LEV were administered in one study. Another study used a combination of IV, oral, rectal and nasogastric tube administration. The route of administration was not indicated in 8 studies. In 22 studies, LEV was given as an adjunct to other AEDs; while it was administered alone in 19 of the studies. Combinations of monotherapy and polytherapy regimen were given in 10 studies.

### Levetiracetam dosing

Levetiracetam dose was titrated in all studies. The median initial dose was 10mg/kg/day [IQR: 10–14]; while the median final dose was 60mg/kg/day [IQR: 40–60]. There was no significant difference (p = 0.986) between the median initial dose in polytherapy (10mg/kg/day [IQR: 10–20]) and monotherapy studies (10mg/kg/day [IQR: 10–10.5]). The median final dose in polytherapy studies (60mg/kg/day [IQR: 60–61.5]) was however significantly higher (p = 0.003) than in monotherapy studies (30mg/kg/day [IQR: 35–52.5]).

### Evidence from Randomised controlled trials

Six RCTs were identified, 4 of which were placebo controlled trials (Table 2). Three of the 6 studies were monotherapy studies comparing LEV treatment with sulthiame (STM), oxcarbazepine (OXC) and placebo respectively. In one study, airway problems (AEs not specified) were significantly more with STM than LEV [12]. None of the other studies reported any significant difference in AEs.

**Table 2:** Data summary for Randomised controlled trials.

Reference	No receiving LEV	Comparator	Age (yrs)	Initial dose (mg/kg/day)	Final dose [mean](mg/kg/d)	Route	Regimen	No of AEs	Follow-up (wks)
Borgraeffe et al, 2013[12]	22	Sulthiame	6–12	10	30	Oral	Monotherapy	64*	24
Fattore et al, 2011[13]	38	Placebo	4–15	10	30[28.5]	Oral	Monotherapy	3	3
Levisohn et al, 2009[14]	64	Placebo	NA	20	60[53.6]	Oral	Polytherapy	145	23
Pina Garza et al, 2009[15]	60	Placebo	<4	20–25	40–50[45.5]	Oral	Polytherapy	26	3
Glauser et al, 2006[16]	101	Placebo	4–16	20	60	Oral	Polytherapy	174	22
Coppola et al, 2006[17]	21	OXC	3–14	5	30	Oral	Monotherapy	3	NA

OXC-oxcarbazepine

* Number of children with AEs not stated.

**Fig 3:**
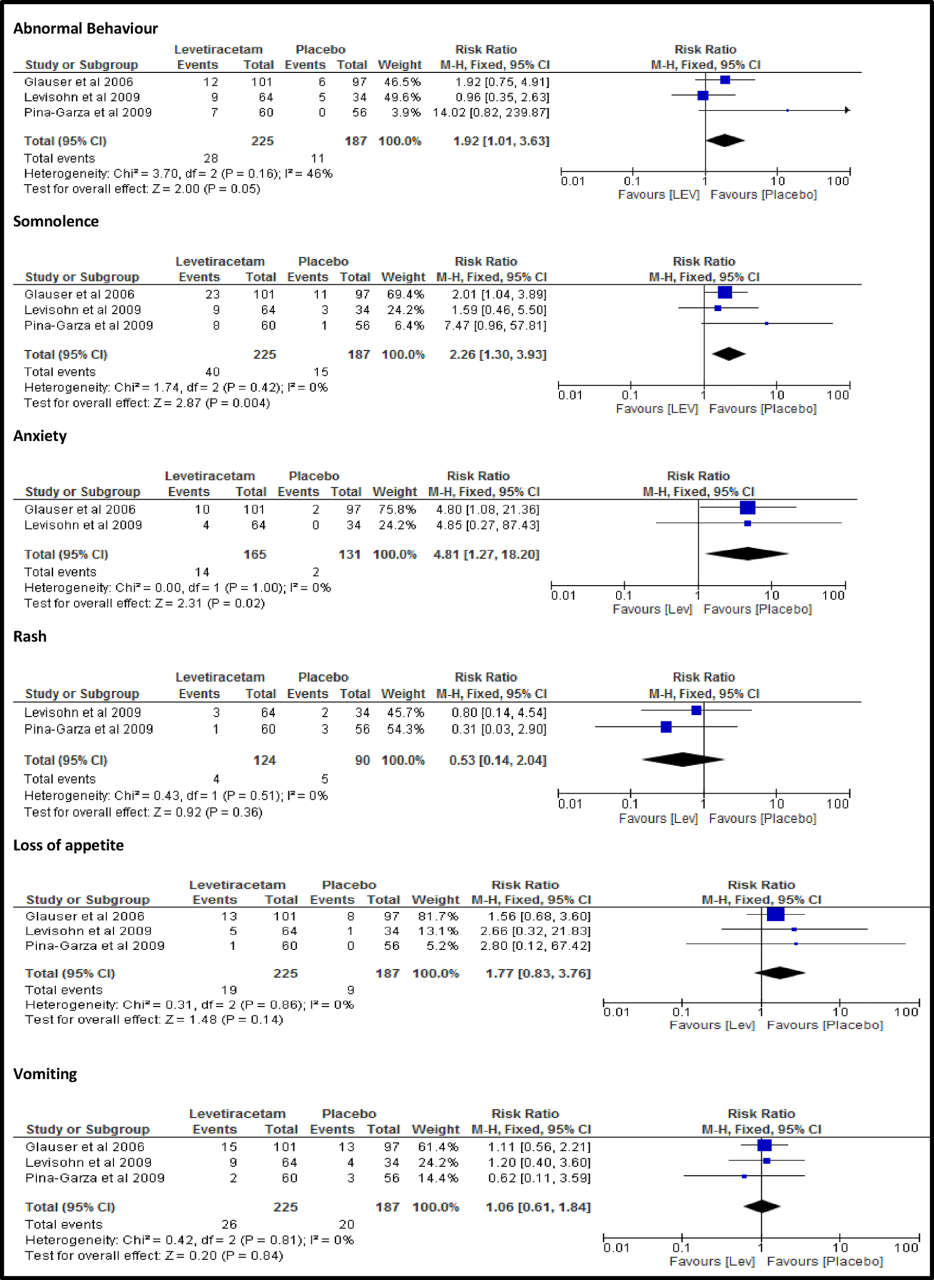
Relative risks of AEs of LEV and placebo.

The three polytherapy studies were add-on placebo controlled studies. Although there was a twofold increase in risk of abnormal behaviour in LEV treated children versus placebo [RR: 1.92 95%CI: 1.01–3.63], this did not reach statistical significance (p = 0.05). Twelve percent of children on LEV developed abnormal behaviours such as: aggression, irritability and hyperactivity; compared to 6% of those on placebo. There was also an increase in the risk of somnolence in LEV treated children compared with placebo [RR: 2.24, 95%CI: 1.29–3.86,p = 0.004], about 18% vs. 8% respectively. About 8.5% of LEV treated children had reports of anxiety compared with 1.5% of placebo, representing a significant increase [RR: 4.81, 95%CI: 1.27–18.20, p = 0.02] (Fig 3). There was no significant difference in the risks of other commonly reported AEs such as loss of appetite, vomiting, dizziness, rash and insomnia (Fig 3).

### Evidence from prospective studies

Prospective studies included prospective cohort studies, RCTs and PK studies and included 1690 children (Tables 2 and 3). 21 of the 30 studies stated the number of children who experienced AEs and included 1120 children. A total of 995 AEs were reported in 525 children (47%). The majority (26%) of the AEs were psychiatric events. Behavioural problems accounted for 64% of all psychiatric events. 10.9% of all AEs were behavioural problems. Common ones included: aggression, irritability and hyperactivity. In several studies, the form of the behavioural problem was not reported. Neurological AEs were also frequently reported, accounting for 24% of AEs. Somnolence was the most common neurological AE (Table 4) occurring in 8.4% of children. Common (≥1/100 to <1/10) neurological events included: headache, dizziness and drowsiness. Vomiting, abdominal pain and diarrhoea were the commonly reported gastrointestinal events. 12% of AEs were gastrointestinal (Table C in S1 Table).

**Table 3:** Prospective cohort studies.

Author (year)	No participants	Age (yrs)	Initial dose (mg/kg/day)	Final dose [mean](mg/kg/d)	Route	Regimen	No of AEs	Follow-up (weeks)
Callenbach et al, 2007[18]	33	4–16	10	60 [22]†	Oral	Polytherapy	139	26
Nakken et al, 2003([19]	44	1–17	10	40	Oral	Polytherapy	11*	32
Kanemura et al, 2013[20]	61	1.3–18	10	60[46.1]	Oral	Polytherapy	2	24
Chhun et al, 2011[21]	102	NA	10	60[31.1]	Oral	Polytherapy	97	24
Kim et al, 2014[22]	55	1.1–18.6	10	20–80[41.9]	Oral	Polytherapy	21	16
Glauser et al, 2002[23]	24	6–12	10	40[36.3]	Oral	Polytherapy	70*	18
Zhang et al, 2014 [24]	105	NA	20	30–40	NA	Polytherapy	2*	NA
Schieman et al, 2012[25]	103	4–16	10	100[50.2]	NA	Polytherapy	196	48
McTague et al, 2012[26]	51	0.3–18	5–30	[14.4]	IV	Monotherapy	3	NA
Weinstock et al, 2013[27]	62	0.1–16	14->40	NA	IV	Monotherapy	32	3
Furwensteches et al, 2010[28]	6	<28days	10	50	Oral	Monotherapy	1	90
Feng et al, 2014[29]	210	1–18	10	60	Oral	Monotherapy	171*	208
Verrotti et al, 2007[30]	21	5–12	NA	NA	Oral	Monotherapy	2	52
Verrotti et al, 2009[31]	12	6–16	10	20.7–45.2	Oral	Monotherapy	4	72
Gao et al, 2008[32]	32	0.7–12	10	40[35]	NA	Monotherapy	11*	NA
Lagae et al, 2005[33]	77	0.5–16	12	62[33]†	Oral	Mixed	12*	20
Dureau et al, 2014[34]	116	<16	NA	NA	Oral	Mixed	15	52
Ng et al, 2010[35]	30	0.5–15	NA	31.1–50.3	IV/Oral	Mixed	11	4
Ramatani et al, 2010[36]	38	<28days	10	45–60	IV	Mixed	2*	52
Li et al, 2011[37]	129	0–16	10	60[29]†	NA	Mixed	97	52

* Number of children with AEs not stated

†median.

**Table 4:** Risk of adverse events occurring in ≥10 children from 30 prospective studies.

System	Adverse Event	Number of patients	Risk per 100 patients	95% Confidence Interval
Psychiatry				
*Behavioural*	Irritability	71	4.2	3.2–5.2
	Abnormal behaviour	52	3.1	2.3–3.9
	Hyperactivity	49	2.9	2.1–3.7
	Aggression	44	2.6	1.9–3.5
*Others*	Dysphoria	32	1.9	1.3–2.6
	Cognitive problems	23	1.4	0.8–2.0
	Anxiety	29	1.7	1.1–2.3
	Learning problem	11	0.7	0.3–1.1
Nervous				
	Somnolence	142	8.4	7.1–9.7
	Headache	64	3.8	2.9–4.7
	Other sleep disorders	29	1.7	1.1–2.3
	Dizziness	21	1.2	0.7–1.7
	Aggravated seizure	16	0.9	0.5–1.4
	Insomnia	14	0.8	0.4–1.2
	Tremor	11	0.7	0.3–1.1
General				
	Loss of appetite	80	4.7	3.7–5.7
	Weakness	64	3.8	2.9–4.7
	Pyrexia	44	2.6	1.9–3.5
	Fatigue	35	2.1	1.4–2.8
Gastro intestinal				
	Vomiting	50	2.9	2.1–3.7
	Abdominal pain	34	2.0	1.3–2.7
	Diarrhoea	28	1.7	1.1–2.3
	Nausea	14	0.8	0.4–1.2
	Gastroenteritis	10	0.6	0.2–1.0
Respiratory				
	Nasopharyngitis	41	2.4	1.7–3.1
	Respiratory tract infection	36	2.1	1.4–2.8
	Cough	24	1.4	0.8–2.0
Skin	Rash	16	0.9	0.5–1.4

There were 7 prospective studies involving 229 paediatric patients where it was possible to determine the number of children with AEs following LEV monotherapy. Fifty one (22%) of these children had at least one AE. There were 10 prospective studies involving 613 paediatric patients on polytherapy and 392 children (64%) had at least one AE. Polytherapy was associated with a significantly higher rate of AEs (p<0.001). There were 82 children who experienced AEs where it was not possible to determine whether they were on polytherapy or monotherapy. The risks of headache, vomiting, hyperactivity and aggression were all lower among monotherapy users (p<0.001). The only AE significantly more common in association with monotherapy was irritability and there was no difference in the occurrence of somnolence between polytherapy and monotherapy users (Table 5).

**Table 5:** Comparison of the most frequent AEs (from 23 prospective studies) among children on polytherapy and monotherapy

Adverse event	Poly [n = 786]	Mono[n = 493]	P value
Headache	54	9	< 0.001*
Vomiting	47	3	< 0.001*
Hyperactivity	33	0	< 0.001*
Aggression	29	3	<0.001*
Pyrexia	35	6	0.001*
Nasopharyngitis	41	0	< 0.001*
Respiratory tract infection	34	0	< 0.001*
Abdominal pain	24	0	< 0.001*
Anxiety	24	0	< 0.001*
Diarrhoea	28	0	< 0.001*
Cough	24	0	< 0.001*
Cognitive problems	19	4	0.04*
Drowsiness	17	1	0.003*
Rash	15	1	0.008*
Nausea	14	0	0.001*
Irritability	26	45	< 0.001*
Somnolence	68	35	0.3
Loss of appetite	39	19	0.4
Weakness	36	33	0.1
ǂ Abnormal behaviour	100	81	0.1
Dizziness	15	3	0.1
Aggravated seizure	11	4	0.4

ǂ includes irritability, aggression, hyperactivity and unclassified abnormal behaviours

* Difference is statistically significant.

### Evidence from retrospective studies

There were 21 retrospective studies (Table A in S1 Table). Similar to reports from prospective studies, both somnolence and abnormal behaviour were the most commonly reported AEs. The majority of the 546 AEs in these studies were psychiatric events (46%). Seventy eight percent of psychiatric events were behavioural problems. The types of behavioural problems were not specified in many of the studies; however, the commonly reported behavioural problems were aggression (3.8/100) and irritability (1.6/100). Neurological events were also frequently reported (34%); while only 3% of AEs were gastrointestinal events (Table B in S1 Table).

### Case reports

There were 16 case reports, with 23 reports of adverse drug reactions (ADRs). The majority of the reported cases were aggravated seizures and psychoses. Other reports included rash, weight loss, depression, autistic regression, acute pancreatitis, elevated alkaline phosphatase, thrombocytopaenia, interstitial nephritis and interstitial lung disease (Table D in S1 Table). The single report of drug interaction was a case of renal failure in a 15 year old boy who received methotrexate for acute lymphoblastic leukaemia [38].

### Treatment discontinuation due to adverse drug reactions

There were 69 reported cases of LEV treatment discontinuation due to ADRs, from a total of 3151 children receiving LEV (2.2%). Forty five of these were from retrospective studies and 24 from prospective studies. 56 of the 1256 children on polytherapy (4.5%) and 7 of the 795 receiving monotherapy (0.9%) discontinued treatment due to toxicity. Six cases of discontinuation were in studies with mixed (polytherapy and monotherapy) treatment. The discontinuation rates were significantly higher on polytherapy than on monotherapy (p <0.001). The majority of discontinuation (33 cases, 48%) was due to behavioural problems. The nature of the behavioural problems was not stated for 22 cases; there were 6 cases of aggression and 5 of irritability. There were 7 cases of somnolence (10%) and 6 cases of seizure aggravation (9%) resulting in treatment discontinuation (Table 6).

**Table 6:** Levetiracetam adverse events leading to treatment discontinuation in prospective and retrospective studies.

Adverse event	Number of patients
Abnormal behaviour	22
Somnolence	7
Aggravated seizure	6
Aggression	6
Irritability	5
Rash	5
Dizziness	3
Insomnia	3
Fatigue	2
Muscle dystrophy	2
Abdominal pain	1
Abnormal reflex	1
Diplopia	1
Headache	1
Loss of appetite	1
Neutropenia	1
Rectal bleeding	1
Speech problem	1
**Total**	**69**

## Discussion

This review shows that behavioural problems and somnolence are the most common adverse events to LEV, with behavioural problems being the most common reasons for treatment discontinuation. Compared with placebo, a twofold increase in the risk of abnormal behaviour was reported in children receiving LEV. This is in line with a previous systematic review, which was focused on only behaviour [39]. Behavioural effects seen with LEV treatment could be positive or negative. Examples of positive effects include: increased energy, vigilance, and activity. Aggression, irritability, hyperactivity and nervousness are frequently observed negative effects. Overall, 2.2% of children receiving LEV had to stop taking the drug due to toxicity. This is similar to the discontinuation rate observed with lamotrigine [40]. When used in combination with another AED, the discontinuation rate was 4.5%.

A systematic review in adults had reported a higher prevalence of anxiety and depression [41]. A prior history of behavioural problem, learning disability and psychiatric conditions can predispose to behavioural decompensation in children on LEV treatment [42]. Behavioural problems associated with LEV have been shown to be worse in epilepsy patients than those taking the drug for cognitive or anxiety disorders, suggesting that epilepsy itself may be a risk factor for abnormal behaviour [41]. Somnolence was reported in about 9% of children in this review. Other common neurological effects include headache and dizziness. A twofold increase in the risk of somnolence was observed in children on LEV treatment compared with those given placebo.

Only two RCTs compared the safety of LEV monotherapy with other AEDs [15, 16]. One other study compared LEV as monotherapy with placebo for two weeks [17]. For ethical reasons, the participants in this study were allowed to exit the study to receive appropriate treatment when a seizure occurred. Although the study was able to establish the short term efficacy of LEV monotherapy, very little information on safety was obtained. There is currently no product license for levetiracetam monotherapy in children less than 16 years old [7]. This is due to the absence of sufficient safety and efficacy data on monotherapy in this age group. Aggregated safety data from 17 prospective studies shows that the risks of AEs were lower with monotherapy treatment than polytherapy. A similar outcome has been reported for AEDs in a previous study [43]. The effect of AED polytherapy on behaviour is complex. Each drug is capable of exerting different behavioural effects. This makes causal attribution of such effects to LEV difficult to establish. Reduction in the number of AEDs or converting to monotherapy treatment generally leads to behavioural improvement [44].

It was not possible to explore the effect of dose on behaviour, because the doses of LEV in children with AEs were not specified in several of the articles. Some authors have suggested that abnormal behaviours in LEV treated patients are idiosyncratic [45]. A previous study among a large cohort of adults who received LEV during preclinical development did not show a significant relationship between dose and behaviour problems [41]. Rapid dose titration rate has however been suggested as a possible risk factor [46].

This systematic review has several limitations. The small number of RCTs included in the meta-analysis reduces the strength of evidence. There were no comparator groups for the prospective cohort studies, hence evidence on comparative safety of LEV was not synthesised from these studies. The quality of retrospective studies was not assessed due to lack of validated quality assessment tools; therefore the quality of evidence generated from these retrospective studies is low. In several of the studies, AEs and not ADRs were reported, therefore the causal relationship between LEV and AEs are unknown. The poor reporting of drug toxicity in RCTs of AEDs in children has been noted [47]. In addition, polytherapy RCTs were all add-on studies, which also made causal attribution difficult. Monotherapy studies in newly diagnosed children with epilepsy are necessary to allow comparison of efficacy and toxicity between individual AEDs [43]. This study is also limited by the lack of information on the reporting methods in the individual studies, which makes it impossible to determine the effect of reporting methods on AE/ADR reporting.

In conclusion, behavioural problems and somnolence were the most prevalent adverse events to LEV and were the most common cause of treatment discontinuation. Children on polytherapy have a greater risk of adverse events than those receiving monotherapy. There is currently insufficient evidence on the safety of LEV in neonates; therefore more studies in this age group are required.

## Supporting Information

S1 Appendix SUMARI quality assessment for prospective cohort. (XLSX)

S2 Appendix PRISMA checklist. (DOC)

S1 Table Additional summary tables for prospective studies, retrospective studies and case reports. (DOCX)
