# Supplementary material for: Safety of Levetiracetam in Paediatrics: A Systematic Review
Source: PLoS One. 2016 Mar 1;11(3):e0149686. doi: 10.1371/journal.pone.0149686 (PMC4773020; doi:10.1371/journal.pone.0149686)
Supplement: S1 Table — (DOCX) [file pone.0149686.s003.docx]

| Author (year) | No participants | Age (yrs) | Initial dose (mg/kg/day) | Final dose [mean](mg/kg/d) | Route | Regimen | No of AEs | Follow-up (wks) | Sponsor |
| --- | --- | --- | --- | --- | --- | --- | --- | --- | --- |
| Tan et al, 2007([1](#_ENREF_1)) | 26 | 1-10 | NA | 13.5-68.5[36.9] | NA | Polytherapy | 1 | 32 | NA |
| Gallentine et al, 2009([2](#_ENREF_2)) | 11 | 2-9 | 15 | 70[30]† | IV/PO/NG/PR | Polytherapy | 1 | NA | Pharma |
| Doumbia et al, 2011([3](#_ENREF_3)) | 42 | 2-19 | 8.5 | 60[37.8] | Oral | Polytherapy | 46 | 12 | NA |
| Elberry et al, 2011([4](#_ENREF_4)) | 22 | 3-19 | 10-15 | NA | Oral | Polytherapy | 2 | 52 | NA |
| Grosso et al, 2008([5](#_ENREF_5)) | 81 | 0.1-4 | 5-10 | 62[41] | Oral | Polytherapy | 38 | 52 | NA |
| Incecik et al, 2012([6](#_ENREF_6)) | 102 | 0.2-11 | 10-20 | 60[41.1] | Oral | Polytherapy | 40 | 24 | None |
| Lee et al 2010([7](#_ENREF_7)) | 130 | NA | 10 | 20-60[47] | Oral | Polytherapy | 11 | 24 | NA |
| Li et al 2010([8](#_ENREF_8)) | 24 | 0-4 | 10 | 65[38.8] | Oral | Polytherapy | 26 | 24 | None |
| Obeid et al, 2010([9](#_ENREF_9)) | 32 | 1-19 | NA | 70-275[146]† | Oral | Polytherapy | 4 | NA | NA |
| Michaelides et al, 2008([10](#_ENREF_10)) | 15 | 0.3-13 | 5-50 | 5-92.5[30.4] | NA | Monotherapy | 9 | NA | NA |
| Perry et al, 2008([11](#_ENREF_11)) | 66 | <16 | NA | NA | Oral | Monotherapy | 32 | 109 | Pharma |
| Abend et al, 2009([12](#_ENREF_12)) | 10 | <14 | 6.5-31 | NA | IV | Monotherapy | 0 | NA | Pharma |
| Standish et al, 2010([13](#_ENREF_13)) | 20 | <16 | NA | 16-98.8[37.5] | IV | Monotherapy | 4 | NA | NA |
| Isguder et al, 2014([14](#_ENREF_14)) | 133 | 0.1-18 | 10 | 20 | IV | Monotherapy | 3 | 32 | NA |
| Khan et al, 2011([15](#_ENREF_15)) | 22 | <28days | 50 | - | IV | Monotherapy | 1 | NA | NA |
| Khurana et al, 2007([16](#_ENREF_16)) | 18 | 2-18 | 14 | 60[25] | NA | Monotherapy | 4 | NA | Pharma |
| Pena-Landin et al, 2014([17](#_ENREF_17)) | 48 | NA | NA | 40 | IV | Mixed | 1 | NA | NA |
| Opp et al, 2005([18](#_ENREF_18)) | 285 | 6-17 | NA | 6-140[47.7] | Oral | Mixed | 204 | 4 | Pharma |
| Peake et al, 2006([19](#_ENREF_19)) | 200 | 0-13 | NA | 8-100[39] | NA | Mixed | 48 | NA | NA |
| Perry et al, 2007([20](#_ENREF_20)) | 122 | <4 | NA | 9-139[35.6] | Oral | Mixed | 49 | 80 | Pharma |
| Koukkari et al, 2004([21](#_ENREF_21)) | 52 | 0.7-16 | 8 | 315 | Oral | Mixed | 22 | NA | Pharma |

Table A in S1 Table: Summary of retrospective studies

| System | Adverse Event | Number of patients | Risk per 100 patient |
| --- | --- | --- | --- |
| Psychiatry |  |  |  |
| *Behaviour* | Abnormal behaviour | 104 | 7.1 |
|  | Aggression | 55 | 3.8 |
|  | Irritability | 24 | 1.6 |
|  | Agitation | 7 | 0.5 |
|  | Hyperactivity | 2 | 0.1 |
|  | others | 3 | 0.2 |
| *Others* | Emotional lability | 23 | 1.6 |
|  | Cognitive problem | 13 | 0.9 |
|  | Anxiety | 10 | 0.7 |
|  | Depression | 7 | 0.5 |
|  | Poor concentration | 1 | 0.1 |
|  |  | **249** | **17.4** |
| Nervous | Somnolence | 116 | 7.9 |
|  | Dizziness | 13 | 0.9 |
|  | Insomnia | 11 | 0.8 |
|  | Sleep disturbance | 11 | 0.8 |
|  | Aggravated seizure | 9 | 0.6 |
|  | Tremor | 7 | 0.5 |
|  | Headache | 6 | 0.4 |
|  | Sedation | 5 | 0.3 |
|  | Ataxia | 4 | 0.3 |
|  | Paraesthesia | 1 | 0.1 |
|  | Diplopia | 1 | 0.1 |
|  |  | **184** | **12.6** |
| General | Loss of appetite | 41 | 2.8 |
|  | Weakness | 11 | 0.8 |
|  | Fatigue | 10 | 0.7 |
|  | Weight loss | 2 | 0.1 |
|  | Hypersalivation | 2 | 0.1 |
|  | Weight gain | 1 | 0.1 |
|  |  | **67** | **4.6** |
| Gastrointestinal | Vomiting | 8 | 0.6 |
|  | Nausea | 2 | 0.1 |
|  | Abdominal pain | 2 | 0.1 |
|  | Constipation | 1 | 0.1 |
|  | Diarrhoea | 1 | 0.1 |
|  | Encorporesis | 1 | 0.1 |
|  | Rectal bleeding | 1 | 0.1 |
|  | Hemorrhagic colitis | 1 | 0.1 |
|  |  | **17** | **1.2** |
| Skin | Rash | 5 | 0.3 |
|  | Acne | 1 | 0.1 |
|  | Pruritus | 1 | 0.1 |
|  | Hypertrichosis | 1 | 0.1 |
|  |  | **8** | **0.6** |
| Respiratory | Respiratory infection | 3 | 0.2 |
|  | Apnoea | 1 | 0.1 |
|  | Hyperpnoea | 1 | 0.1 |
|  |  | **5** | **0.3** |
| Haematology | Leucopaenia | **3** | **0.2** |
| Musculoskeletal | Movement disorder | **3** | **0.2** |
| Others | Others | **10** | **0.7** |

Table B in S1 Table: Risk of adverse events occurring in children from retrospective studies

Table C in S1 Table: Risk of adverse events occurring in children from prospective studies

| System | Adverse Event | Number of patients | Risk per 1000 patients |
| --- | --- | --- | --- |
| Psychiatry |  |  |  |
| *Behavioural* | Irritability | 71 | 4.4 |
|  | Abnormal behaviour | 52 | 3.2 |
|  | Hyperactivity | 49 | 3.0 |
|  | Aggression | 44 | 2.7 |
|  | Restlessness | 7 | 0.4 |
| *Others* | Dysphoria | 32 | 1.9 |
|  | Cognitive problems | 23 | 1.4 |
|  | Anxiety | 29 | 1.8 |
|  | Learning problem | 11 | 0.7 |
|  | Emotional lability | 9 | 0.6 |
|  | Personality change | 8 | 0.5 |
|  | Poor concentration | 7 | 0.4 |
|  | Mood disturbance | 4 | 0.3 |
|  | Psychosis | 1 | 0.1 |
|  |  | **347** | **21.3** |
| Nervous | Somnolence | 142 | 8.7 |
|  | Headache | 64 | 3.9 |
|  | Other sleep disorders | 29 | 1.8 |
|  | Dizziness | 21 | 1.3 |
|  | Aggravated seizure | 16 | 1.0 |
|  | Insomnia | 14 | 0.9 |
|  | Tremor | 11 | 0.7 |
|  | Ataxia | 8 | 0.5 |
|  | Sedation | 3 | 0.2 |
|  | Hypotonia | 2 | 0.1 |
|  | Abnormal reflex | 1 | 0.1 |
|  | Others | 13 | 0.8 |
|  |  | **324** | **19.9** |
| General | Loss of appetite | 80 | 4.9 |
|  | Weakness | 64 | 3.9 |
|  | Pyrexia | 44 | 2.7 |
|  | Fatigue | 35 | 2.1 |
|  | Weight loss | 7 | 0.4 |
|  | Weight gain | 6 | 0.4 |
|  | Others | 83 | 5.1 |
|  |  | **319** | **19.6** |
| Gastro intestinal | Vomiting | 50 | 3.1 |

|  | Abdominal pain | 34 | 2.1 |
| --- | --- | --- | --- |
|  | Diarrhoea | 28 | 1.7 |
|  | Nausea | 14 | 0.9 |
|  | Gastroenteritis | 10 | 0.6 |
|  | Constipation | 8 | 0.5 |
|  | Others | 10 | 0.6 |
|  |  | **154** | **9.4** |
| Respiratory | Nasopharyngitis | 41 | 2.5 |
|  | Respiratory tract infection | 36 | 2.2 |
|  | Cough | 24 | 1.5 |
|  | Others | 36 | 2.2 |
|  |  | **137** | **8.4** |
| Skin | Rash | 16 | 1.0 |
|  | Hair loss | 4 | 0.3 |
|  | Pruritus | 4 | 0.3 |
|  |  | **24** | **1.5** |
| Cardiovascular | Hypotension | 5 | 0.3 |
|  | Bradycardia | 3 | 0.2 |
|  | Tachycardia | 1 | 0.1 |
|  |  | **9** | **0.6** |
| Musculoskeletal | Muscle dystrophy | 8 | 0.5 |
|  | Others | 4 | 0.3 |
|  |  | **12** | **0.7** |
| Haematological | Leucopoenia | 4 | 0.3 |
|  | Neutropenia | 1 | 0.1 |
|  | Anaemia | 1 | 0.1 |
|  |  | **6** | **0.4** |
| Urinary | Incontinence | 1 | 0.1 |
|  | Oliguria | 1 | 0.1 |
|  |  | **2** | **0.1** |
| Hepatobiliary | Elevated enzymes | **1** | **0.1** |
| Others | Others | **12** | **0.7** |

Table D in S1 Table: Summary of case reports

| Reference | Adverse reaction | Age (yrs) | Gender | Dose | Median days of onset | Co-medication |
| --- | --- | --- | --- | --- | --- | --- |
| Koul et al, 2008([22](#_ENREF_22)) | Weight loss | 4 | M | 37mg/kg/day | NA | Clonazepam, PHT,VPA |
|  | Weight loss | 1 | F | NA | NA | Clonazepam, LTG,VPA |
| Tamarelle et al, 2008([23](#_ENREF_23)) | Depression | 5 | M | NA | 21 | LTG, VPA |
| Peer et al, 2009([24](#_ENREF_24)) | Thrombocytopaenia | 6 | M | NA | 35 | NA |
| Caraballo et al, 2010([25](#_ENREF_25)) | Aggravated seizure | 10 | F | 43mg/kg/day | NA | LTG |
|  | Aggravated seizure | 10 | M | 1000mg/day | NA | CLB, TPM |
| Isoda et al, 2013([26](#_ENREF_26)) | Aggravated seizure | 9 | M | 250mg/day | NA | CLB, TPM,LTG |
| Liu et al, 2012([27](#_ENREF_27)) | Aggravated seizure | 8 | M | 6mg/kg/day | NA | None |
|  | Aggravated seizure | 10 | M | 5mg/kg/day | NA | None |
|  | Aggravated seizure | 10 | M | 7mg/kg/day | NA | None |
| Kroll-Seger et al 2006([28](#_ENREF_28)) | Aggravated seizure | 3 | M |  | NA | None |
| Almeida et al, 2013([29](#_ENREF_29)) | Acute pancreatitis | 17 | M | 250mg/day | 15 | None |
| Koklu et al, 2014([30](#_ENREF_30)) | Rash | 0.1 | M | 10mg/kg/day | 1 | None |
| Zaki et al, 2014([31](#_ENREF_31)) | Psychosis | 11 | F | 40mg/kg/day | 1 | None |
| Kossof et al, 2001([32](#_ENREF_32)) | Psychosis | 5 | F | 25mg/kg/day | 14 | None |
|  | Psychosis | 13 | M | 16mg/kg/day | 90 | None |
|  | Psychosis | 16 | F | 15mg/kg/day | 2 | CBZ |
|  | Psychosis | 17 | F | 33mg/kg/day | 30 | CBZ |
| Youroukos et al, 2003([33](#_ENREF_33)) | Psychosis | 12 | F | 60mg/kg/day | 10 | VPA |
| Scot et al, 2007 ([34](#_ENREF_34)) | Interstitial lung disease | 9 | F | - | 730 | None |
| Xiong et al 2012 ([35](#_ENREF_35)) | Elevated alkaline phosphatase | 0.8 | F | 27.8mg/kg/day | 150 | None |
| Camacho et al 2012 ([36](#_ENREF_36)) | Autistic regression | 6 | F | 53mg/kg/day | 180 | None |
| Hurwitz et al 2009 ([37](#_ENREF_37)) | Interstitial nephritis | 17 | F | 10mg/kg/day | 10 | None |

Table E in S1 Table: Pharmacokinetic studies

| Author (year) | No receiving LEV | Comparator | Age (yrs) | Initial dose (mg/kg/day) | | Final dose [mean](mg/kg/d) | | Route | Regimen | No of AEs | Follow-up (wks) | Sponsor |
| --- | --- | --- | --- | --- | --- | --- | --- | --- | --- | --- | --- | --- |
| Fountain et al, 2007([38](#_ENREF_38)) | 21 | - | 4-12 | | 20 | | 60 | Oral | Polytherapy | 20 | 12 | NA |
| Glauser et al, 2007([39](#_ENREF_39)) | 13 | - | 0.2-4 | | 20 | | NA | Oral | Polytherapy | 4 | 2 | institutional &pharma |
| Sharpe et al, 2012([40](#_ENREF_40)) | 18 | - | <28days | | 20-40 | | NA | IV | Monotherapy | 8 | NA | Institutional |
| Merhar et al, 2011([41](#_ENREF_41)) | 21 | - | <28days | | 14.4-39.9 | | - | IV | Monotherapy | ** | 1 day | Institutional |

References

1. Tan MJ, Appleton RE. Efficacy and tolerability of levetiracetam in children aged 10 years and younger: a clinical experience. Seizure. 2004;13(3):142-5.

2. Gallentine WB, Hunnicutt AS, Husain AM. Levetiracetam in children with refractory status epilepticus. Epilepsy Behav. 2009;14(1):215-8.

3. Doumbia-Ouattara M, Bourel-Ponchel E, Le Moing A, Querne L, Delignières A, de Broca A, et al. [Experience with levetiracetam in the treatment of childhood refractory epilepsy]. Archives de pediatrie: organe officiel de la Societe francaise de pediatrie. 2012;19(1):3-8.

4. Elberry AA, Felemban RK, Hareeri RH, Kurdi SM. Efficacy and safety of levetiracetam in pediatric epilepsy. Saudi Pharmaceutical Journal. 2012;20(1):81-4.

5. Grosso S, Cordelli D, Franzoni E, Coppola G, Capovilla G, Zamponi N, et al. Efficacy and safety of levetiracetam in infants and young children with refractory epilepsy. Seizure. 2007;16(4):345-50.

6. Incecik F, Hergüner MO, Altunbasak S. The efficacy and side effects of levetiracetam on refractory epilepsy in children. Journal of Pediatric Neurosciences. 2012;7(1):19.

7. Lee YJ, Kang H-C, Kim HD, JS L. Efficacy and safety of adjunctive levetiracetam therapy in pediatric intractable epilepsy. Pediatr Neurol. 2010;42:86-92.

8. Li S, Cao J, Xiao N, Cai F. Efficacy and safety of levetiracetam as an add-on therapy in children aged less than 4 years with refractory epilepsy. J Child Neurol. 2009.

9. Obeid M, Pong AW. Efficacy and tolerability of high oral doses of levetiracetam in children with epilepsy. Epilepsy Res. 2010;91(1):101-5.

10. Michaelides C, Thibert RL, Shapiro MJ, Kinirons P, John T, Manchharam D, et al. Tolerability and dosing experience of intravenous levetiracetam in children and infants. Epilepsy Res. 2008;81(2):143-7.

11. Perry S, Holt P, Benatar M. Levetiracetam versus carbamazepine monotherapy for partial epilepsy in children less than 16 years of age. J Child Neurol. 2008.

12. Abend NS, Monk HM, Dlugos DJ. Intravenous levetiracetam in critically ill children with status epilepticus or acute repetitive seizures. Pediatr Crit Care Med. 2009;10(4):505-10.

13. Standish JC, Hilmas E, Falchek SJ. Levetiracetam for the treatment of pediatric status epilepticus: A case series. Journal of Pediatric Neurology. 2011;9(2):195-201.

14. İşgüder R, Güzel O, Ağın H, Yılmaz Ü, Akarcan SE, Çelik T, et al. Efficacy and Safety of IV Levetiracetam in Children With Acute Repetitive Seizures. Pediatr Neurol. 2014;51(5):688-95.

15. Khan O, Chang E, Cipriani C, Wright C, Crisp E, Kirmani B. Use of intravenous levetiracetam for management of acute seizures in neonates. Pediatr Neurol. 2011;44(4):265-9.

16. Khurana DS, Kothare SV, Valencia I, Melvin JJ, Legido A. Levetiracetam monotherapy in children with epilepsy. Pediatr Neurol. 2007;36(4):227-30.

17. Peña-Landín D, Ruíz G. Experiencia clínica con levetiracetam intravenoso en 48 pacientes pediátricos para el manejo de crisis convulsivas agudas y descontrol de epilepsia.

18. Opp J, Tuxhorn I, May T, Kluger G, Wiemer-Kruel A, Kurlemann G, et al. Levetiracetam in children with refractory epilepsy: a multicenter open label study in Germany. Seizure. 2005;14(7):476-84.

19. Peake D, Mordekar S, Gosalakkal J, Mukhtyar B, Buch S, Crane J, et al. Retention rate of levetiracetam in children with intractable epilepsy at 1 year. Seizure. 2007;16(2):185-9.

20. Perry MS, Benatar M. Efficacy and tolerability of levetiracetam in children younger than 4 years: a retrospective review. Epilepsia. 2007;48(6):1123-7.

21. Koukkari MW, Guarino EJ. Retrospective study of the use of levetiracetam in childhood seizure disorders. J Child Neurol. 2004;19(12):944-7.

22. Koul R, Al-Futaisi A. Levetiracetam induced weight loss in two children. Journal of Pediatric Neurology. 2008;6(3):257-60.

23. Tamarelle C, Pandit F, Mazarati A, Riquet A, Vallée L, Auvin S. Levetiracetam-induced depression in a 5-year-old child with partial epilepsy. Seizure. 2009;18(3):235-6.

24. Peer MB, Prabhakar P. Thrombocytopenia as an adverse effect of levetiracetam therapy in a child. Neuropediatrics. 2009;40(5):243.

25. Caraballo RH, Cersósimo R, De los Santos C. Levetiracetam-induced seizure aggravation associated with continuous spikes and waves during slow sleep in children with refractory epilepsies. Epileptic Disord. 2010;12(2):146-50.

26. Isoda K. Nonconvulsive status epilepticus in a child: case report. Reactions. 2012;1430:1.

27. Liu Y-H, Wang X-L, Deng Y-C, Zhao G. Levetiracetam-associated aggravation of myoclonic seizure in children. Seizure. 2012;21(10):807-9.

28. Kröll-Seger J, Mothersill IW, Novak S, Sälke-Kellermann RA, Krämer G. Levetiracetam-induced myoclonic status epilepticus in myoclonic-astatic epilepsy: a case report. Epileptic Disord. 2006;8(3):213-8.

29. Almeida DM, Jean MR, Chystsiakova A, Monahan E, Oliveira SB, Monteiro IM. Levetiracetam-Associated Acute Pancreatitis in an Adolescent With Autism: A Case Report. Pancreas. 2013;42(1):177-8.

30. Koklu E, Ariguloglu EA, Koklu S. Levetiracetam-induced anaphylaxis in a neonate. Pediatr Neurol. 2014;50(2):192-4.

31. Zaki SA, Gupta S. Levetiracetam-induced acute psychosis in a child. Indian J Pharmacol. 2014;46(3):341.

32. Kossoff EH, Bergey GK, Freeman JM, Vining EP. Levetiracetam psychosis in children with epilepsy. Epilepsia. 2001;42(12):1611-3.

33. Youroukos S, Lazopoulou D, Michelakou D, Karagianni J. Acute psychosis associated with levetiracetam. Epileptic Disord. 2003;5(2):117-9.

34. Newsome SD, Xue LY, Jennings T, Castaneda GY. Levetiracetam-induced diffuse interstitial lung disease. J Child Neurol. 2007;22(5):628-30.

35. Xiong N, Hou L, Lu N, Mohamed AA, Wang T, Huang Y. Probable levetiracetam-related serum alkaline phosphatase elevation. BMC neurology. 2012;12(1):97.

36. Camacho A, Espín JC, Nuñez N, Simón R. Levetiracetam-Induced Reversible Autistic Regression. Pediatr Neurol. 2012;47(1):65-7.

37. Hurwitz KA, Ingulli EG, Krous HF. Levetiracetam induced interstitial nephritis and renal failure. Pediatr Neurol. 2009;41(1):57-8.

38. Fountain NB, Conry JA, Rodríguez-Leyva I, Gutierrez-Moctezuma J, Salas E, Coupez R, et al. Prospective assessment of levetiracetam pharmacokinetics during dose escalation in 4-to 12-year-old children with partial-onset seizures on concomitant carbamazepine or valproate. Epilepsy Res. 2007;74(1):60-9.

39. Glauser TA, Mitchell WG, Weinstock A, Bebin M, Chen D, Coupez R, et al. Pharmacokinetics of levetiracetam in infants and young children with epilepsy. Epilepsia. 2007;48(6):1117-22.

40. Sharpe CM, Capparelli EV, Mower A, Farrell MJ, Soldin SJ, Haas RH. A seven-day study of the pharmacokinetics of intravenous levetiracetam in neonates: marked changes in pharmacokinetics occur during the first week of life. Pediatric research. 2012;72(1):43-9.

41. Merhar SL, Schibler KR, Sherwin CM, Meinzen-Derr J, Shi J, Balmakund T, et al. Pharmacokinetics of levetiracetam in neonates with seizures. The Journal of pediatrics. 2011;159(1):152-4. e3.
